# Case report: Caplacizumab without plasma exchange for severe thrombotic thrombocytopenic purpura

**DOI:** 10.3389/fddev.2026.1894135

**Published:** 2026-08-28

**Authors:** Sarah Maryon Hayes, Daniel F. Pease, Christine M. Wiese, Marshall Mazepa

**Affiliations:** 1 North Memorial Health, Cancer Center, Robbinsdale, MN, United States; 2 Division of Hematology, Oncology and Transplantation, University of Minnesota – Department of Medicine, Minneapolis, MN, United States

**Keywords:** caplacizumab, case report, Jehovah’s Witness, plasma exchange, thrombotic thrombocytopenic purpura

## Abstract

A 45-year-old female Jehovah’s Witness presented with transient expressive aphasia, blurred vision, and dizziness for several hours. A complete blood count (CBC) showed anemia (hemoglobin: 6.3 g/dL), thrombocytopenia (platelet count: 21 × 10^3^/µL), and undetectable haptoglobin. The peripheral smear revealed schistocytes, confirming microangiopathic hemolytic anemia; immune-mediated thrombotic thrombocytopenic purpura (iTTP) was confirmed with an ADAMTS13 level of less than 5% and a positive ADAMTS13 inhibitor screen. Treatment with high-dose corticosteroids was urgently initiated, but the patient and her husband declined plasma exchange based on their beliefs as Jehovah’s Witnesses. The patient acutely decompensated, requiring intubation, with worsening of laboratory values (hemoglobin: 3.8 g/dL; platelet count: 9 × 10^3^/µL), elevated serum troponin, and renal and hepatic compromise. In addition to rituximab and intravenous immune globulin, caplacizumab therapy was undertaken. The patient’s condition improved clinically, but from a laboratory value perspective, 1 month after caplacizumab initiation, her ADAMTS13 level remained less than 5%. Further immunosuppression with intravenous cyclophosphamide was initiated, which resulted in complete normalization of ADAMTS13 activity. This is the first report of caplacizumab use in a Jehovah’s Witness patient with TTP with severe neurological compromise to the point of necessitating intubation and with this degree of anemia. With the eventual addition of cyclophosphamide therapy, we observed complete recovery of this critically ill patient without the use of plasma exchange in the treatment course.

## 1 Introduction

Thrombotic thrombocytopenic purpura (TTP) is an uncommon and potentially fatal form of thrombotic microangiopathy (TMA), characterized by a pentad of findings including microangiopathic hemolytic anemia (MAHA), severe thrombocytopenia, neurological abnormalities, renal injury, and fever. Immune-mediated TTP results from severe deficiency of ADAMTS13 activity and the presence of autoantibodies that inhibit or clear ADAMTS13 (Chiasakul and Cuker, 2018; Joly et al., 2017). When ADAMTS13 is inhibited from cleaving VWF multimers, the multimers accumulate, inducing platelet aggregation and adhesion, leading to microthrombi and red blood cell shearing. This process results in MAHA, thrombocytopenia, and end-organ ischemia. Timely recognition and urgent treatment of iTTP are critical.

Multiple factors may predispose an individual to developing iTTP, including environment, female sex, obesity, and ethnicity/race. Certain HLA haplotypes are associated with iTTP. The HLA-DRB1*04 is overrepresented in the Caucasian population and appears to have a protective effect in this population (Sukumar et al., 2020). The HLA-DRB1*04 is markedly reduced in populations of African ancestry, which possibly accounts for the eightfold increased incidence of iTTP among Black individuals in the United States (Sukumar et al., 2020). Notably, the incidence of iTTP is higher in women and African-American individuals (Reese et al., 2013).

Jehovah’s Witness patients view Bible passages as ruling out transfusion of whole blood, red blood cells, white blood cells, platelets, and plasma; however, “The religious understanding of Jehovah’s Witnesses does not absolutely prohibit the use of fractionated blood products (such as albumin, clotting factors, fibrinogen, and hemoglobin solutions). Each witness must decide whether he can accept immunoglobulins or serums made with a blood fraction” (Religious and Ethical Position on Medical Therapy and Related Matters, 2025). Commonly employed strategies for the treatment of anemia in Jehovah’s Witness patients include the use of erythropoiesis-stimulating agents, thrombopoietin agents, tranexamic acid, minimizing blood draws, and supplementation with intramuscular cyanocobalamin, intravenous folic acid, and intravenous iron.

## 2 Case description

**FIGURE 1:**
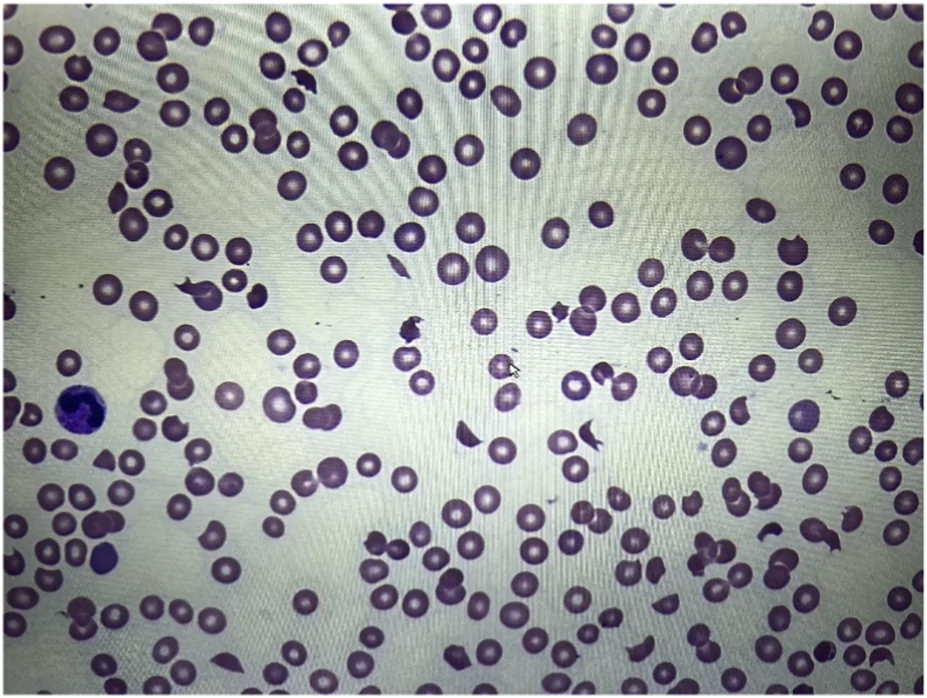
Peripheral smear revealing schistocytes.

A 45-year-old African–American female Jehovah’s Witness with a history of migraines presented to the Emergency Department with several hours of transient expressive aphasia, blurred vision, and dizziness. Initial laboratory work showed a hemoglobin (Hgb) level of 6.3 g/dL, a platelet count (Plt) of 21 × 10^3^/uL, normal coagulation studies, elevated serum troponin levels, a serum creatinine level of 1.1 mg/dL, a total bilirubin level of 1.4 mg/dL (with an indirect bilirubin level of 1.2 mg/dL), an LDH level of 1,078 U/L, and an undetectable haptoglobin level. The patient’s initial blood pressure readings over the first several days were 126/80, 109/78, 121/90, and 133/98 mmHg, respectively, ruling out malignant hypertension. Her urine was noted to be brown-colored. A brain MRI was negative for infarct, dissection, or mass. Clinical suspicion of thrombotic thrombocytopenic purpura (TTP) was raised in the Emergency Department, and hematology was urgently consulted. The peripheral smear revealed schistocytes (Figure 1), confirming microangiopathic hemolytic anemia. The PLASMIC score was calculated to be high at 6 points, suggesting a high likelihood of iTTP (Bendapudi et al., 2017).

**FIGURE 2:**
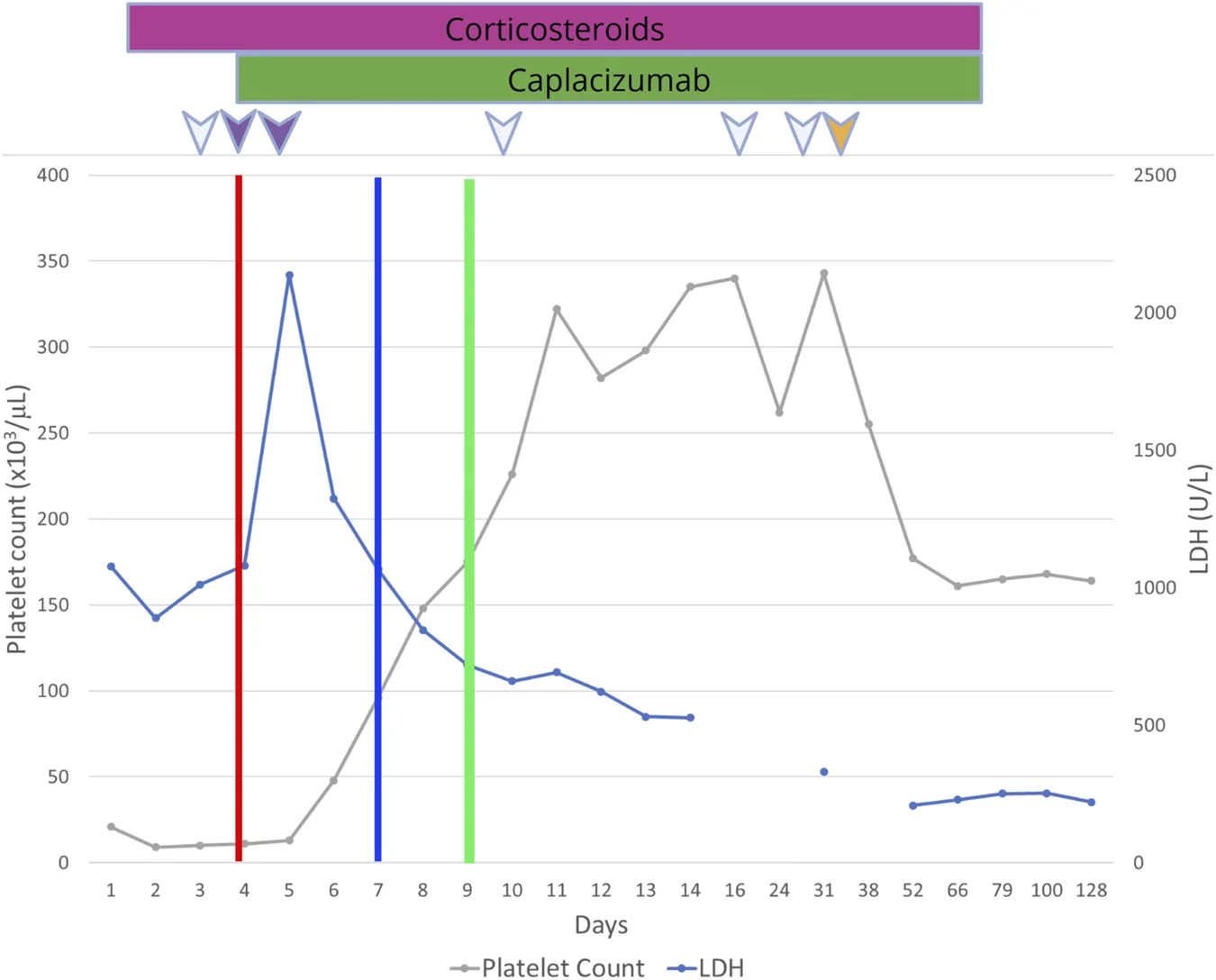
Rapid improvement in iTTP with caplacizumab without PLEX: Corticosteroids were started at diagnosis (Day 1, pink horizontal bar), and rituximab was initiated on Day 3 (light blue arrows); however, the patient deteriorated. On Day 4, when she became critically ill (red vertical line), IVIG (purple arrows) and caplacizumab (green horizontal bar) were started. By Day 7, her neurological symptoms completely normalized (blue vertical line), and by Day 9, she was in clinical remission (green vertical line). To achieve ADAMTS13 remission, she required additional immunosuppression with cyclophosphamide (orange arrow), and then caplacizumab and corticosteroids were stopped. Created with Biorender.com.

Treatment was initiated in the Emergency Department (Figure 2) with intravenous methylprednisolone 1,000 mg daily. The immediate initiation of plasma exchange (PLEX) was strongly recommended by the hematology consult team, and nephrology was consulted for PLEX administration. The patient and her husband declined PLEX based on their beliefs as Jehovah’s Witnesses.

On Day 2, the ADAMTS13 level returned at less than 5% with a positive ADAMTS13 inhibitor screen (Bethesda titer: 4.2 BU/mL), confirming the diagnosis of iTTP. The recommendation was made to initiate intravenous immunoglobulin (IVIg) therapy and plasmapheresis with albumin exchange, both of which were declined by the patient. On Day 3, rituximab 375 mg/m^2^ was administered intravenously. Urgent acquisition of caplacizumab was initiated. Her platelet count decreased to 10 × 10^3^/uL, and other laboratory values did not improve. Unfortunately, on Day 4, she acutely decompensated, becoming unresponsive and requiring intubation and transfer to the intensive care unit. Hemoglobin decreased to 5.4 g/dL.

Given the severity of this patient’s presentation (nadir Hgb: 3.8 g/dL, nadir Plt: 9 × 10^3^/uL, neurological compromise, elevated serum troponin, and renal and hepatic compromise), the hematologist elected to initiate caplacizumab therapy immediately after it was delivered on Day 4. Standard dosing of 11 mg intravenously followed by 11 mg subcutaneously daily was given. A trial of plasmapheresis with albumin replacement was attempted. Shortly after initiation, the plasmapheresis machine began alarming due to the patient’s critically low hematocrit (serum Hct: 11%–16%); the effluent was very turbid with hemolytic products. Additionally, she began oozing significantly from the access site, and the continued use of albumin as a replacement fluid posed a risk of increasing bleeding complications. Thus, attempts at plasmapheresis with albumin were terminated. The patient’s husband also consented to IVIg, which was administered at 1,000 mg/kg daily for two doses. Laboratory analysis was conducted once daily using pediatric tubes to minimize iatrogenic blood loss.

On Day 5, folic acid dosing was increased to 5 mg intravenously daily, and epoetin alfa 20,000 units subcutaneously daily was initiated. Total bilirubin and haptoglobin levels improved. On Day 6, her platelet count improved to 48 × 10^3^/uL, her haptoglobin level increased to 10 mg/dL, her neurological status rapidly improved, and she was successfully extubated. Corticosteroids were adjusted to methylprednisolone at a dose of 1 mg/kg daily. On Day 7, her neurological symptoms had completely resolved. She achieved clinical remission on Day 9 (two consecutive days with a normal platelet count and a continued downtrending of lactate dehydrogenase) after only six doses of caplacizumab without PLEX, and her hemoglobin increased to 6.0 g/dL. Other laboratory parameters also improved: LDH decreased to 661 U/L, total bilirubin fell to 0.7 mg/dL, and haptoglobin increased to 9 mg/dL. On Day 14, the patient was discharged from the hospital on 11 mg of subcutaneous caplacizumab daily and tapering corticosteroids; she remained on prednisone 1 mg/kg (80 mg) daily, with a gradual taper off over approximately 6 weeks: 60 mg daily for 1 week, then 50 mg daily for 1 week, then 40 mg daily for 1 week, followed by 30 mg daily for 5 days, then 20 mg daily for 5 days, 10 mg daily for 5 days, 5 mg daily for 1 week, and then 5 mg every other day for 1 week.

Despite the use of corticosteroids, weekly rituximab for four doses, and IVIg, the patient’s ADAMTS13 level remained below 5% 1 month after caplacizumab initiation. Further immunosuppression with intravenous cyclophosphamide was initiated (Beloncle et al., 2012). Her ADAMTS13 level normalized to 79% following one dose of intravenous cyclophosphamide (600 mg/m^2^), and caplacizumab was discontinued on Day 50 of therapy. No adverse events, such as bleeding, were observed during the course of caplacizumab. Cyclophosphamide was discontinued after four doses due to the complete normalization of laboratory values (ADAMTS13 > 100%, Plt: 164 × 10^3^/uL, Hgb: 11.9 g/dL, LDH: 221 U/L) and the absence of recurrence of neurological symptoms. She remains in clinical and ADAMTS13 remission.

## 3 Discussion

Caplacizumab, FDA-approved in combination with PLEX for the treatment of iTTP, is a humanized immune globulin that targets the A1 domain of VWF, resulting in the inhibition of platelet binding and prevention of the formation of microvascular thrombi. Caplacizumab therapy for patients unable or unwilling to proceed with plasmapheresis for the treatment of iTTP has been described in the literature, first in 2019, when a patient with iTTP refused plasma exchange for religious reasons (Chander et al., 2019). Several case reports of successful use of caplacizumab without PLEX in the management of iTTP have been published since (Sukumar et al., 2020; Völker et al., 2020; Spencer et al., 2022; Desai et al., 2021; Cardesa-Salzmann et al., 2022; Irani et al., 2020; Deucher et al., 2022; Warr, 2022). A recent report on 42 iTTP episodes in 41 patients from Germany and Austria found that, with early initiation of caplacizumab without PLEX, there was no difference in outcomes (e.g., time to remission or death) compared with a similar cohort treated with plasma exchange (Kühne et al., 2024). Similarly, the results of a Phase 3, single-arm, open-label trial [MAYARI, NCT05468320] evaluating caplacizumab for iTTP without the use of PLEX were recently completed. They showed that 93.5% (43/46) of participants achieved remission without PLEX and 97.8% (45/46) achieved remission, adding evidence that caplacizumab without PLEX is safe and likely very effective (Coppo et al., 2025).

However, this is the first report of caplacizumab use in a patient with iTTP with severe neurological compromise to the point of necessitating intubation and with severe anemia. Caplacizumab is known to bind to the A1 domain of VWF but not necessarily displace platelets already bound to VWF. Hence, the drug is more akin to an anticoagulant than a thrombolytic. It is likely that, to resolve established platelet-rich thrombi, ADAMTS13 is still required (Ulrichts et al., 2011). Thus, for patients with milder symptoms, less organ damage, and presumably less microvascular thrombotic burden, it is not surprising that caplacizumab alone can be sufficient to resolve the acute episode without the addition of exogenous plasma as a source of ADAMTS13. In this case, the patient’s rapid resolution of severe neurological impairment within 48 h was remarkable for this reason and suggests that caplacizumab alone without PLEX may be sufficient to reduce organ damage and lead to clinical remission without plasma.

Given the high cost of caplacizumab therapy, its role in the management of iTTP is debated. In our case, early initiation of caplacizumab was pivotal in preventing further end-organ damage and achieved rapid improvement in platelet count. The ISTH guidelines for the treatment of iTTP state that caplacizumab should be initiated, where possible, in the early phase of an acute iTTP episode when diagnosis is confirmed (Zheng et al., 2020). The patient in this report started caplacizumab therapy on Day 4 of therapy (immediately after the drug became available); starting caplacizumab therapy even earlier may have prevented such a severe course, as iTTP is a time-dependent disease. The patient reported adherence to home caplacizumab therapy with no untoward effects. We continued caplacizumab until Day 50, when the ADAMTS13 level normalized.

In summary, this case illustrates the successful treatment of a Jehovah’s Witness with severe TTP without the use of therapeutic PLEX, achieved with caplacizumab to prevent microvascular thrombosis and, ultimately, cyclophosphamide for sustained immune suppression. Further studies will elucidate the role of caplacizumab, without PLEX, for the treatment of TTP.

## Data Availability

The raw data supporting the conclusions of this article will be made available by the authors, without undue reservation.
